# Comment on “Changes in iPSC-Astrocyte Morphology Reflect Alzheimer’s Disease Patient Clinical Markers”

**DOI:** 10.1093/stmcls/sxaf030

**Published:** 2025-05-13

**Authors:** Leepy Paudel

**Affiliations:** Department of Stem Cell and Regenerative Medicine, Johns Hopkins University, Baltimore, MD 21218, USA

Dear editors,

The study, “Changes in iPSC-Astrocyte Morphology Reflect Alzheimer’s Disease Patient Clinical Markers,” demonstrates one of the many uses of human induced pluripotent stem cells (iPSCs). Since iPSCs can replicate disease behaviors, the authors investigate how iPSC-derived astrocytes might be used to model Alzheimer’s disease (AD).

After culturing the iPSCs of AD patients, the authors discovered that astrocytes respond to harmful stimuli like β-amyloid (Aβ) oligomers, which are the hallmark of AD. They found that astrocytes of patients who had lower levels of the inflammatory biomarker YKL-40 in their cerebrospinal fluid experienced major structural alterations. Whereas, changes were found to be less noticeable in patients with greater levels of YKL-40. This was associated with the presence of the apolipoprotein E (APOE) ε4/ε4 genotype, a known genetic risk factor for AD. Hence, YKL-40s function is an essential biomarker for glial activation in AD.^1^

A study used a similar technique and created a model of astrocytes derived from iPSCs from both healthy people and AD patients in order to identify functional differences. The authors highlighted the role of astrocytes in AD pathology and its uses for medicine and drug screening. The astrocytes obtained from AD patients showed a number of abnormal behaviors, including altered inflammatory responses and were unable to sustain neurons. This demonstrates how AD can affect the brain’s supporting glial cells in addition to neurons.^2^

The study’s use of deep learning methods to examine astrocyte morphology was another interesting technique used by the authors. They classified several astrocyte phenotypes according to how they responded to Aβ exposure by using similarity learning algorithms. This method provides a better way of measuring the cellular alterations observed in neurodegenerative illnesses by removing any user bias, and therefore, could revolutionize drug screening.^1^

Other studies have also highlighted the potential use of deep learning methods to investigate astrocyte morphology in neurodegenerative illnesses. Their results also demonstrate that these methods are important to better understand the cellular alterations in AD and related disorders.^3^

This study is important because it shows us that the iPSC-derived astrocytes could be a potent tool for personalized treatment. Because astrocytes are unique to each patient, they offer important information about how each person’s illness may develop and how they might react to different therapies. These astrocytes can also recreate reactive morphologies in response to neurotoxic stimuli.^4^ This is a huge opportunity for Alzheimer’s research.

In conclusion, this study provides valuable insights into the role of iPSC-derived astrocytes in AD research. It opens the door for more accurate patient categorization and focused treatments by incorporating deep learning analytics. These models’ capacity to capture patient-specific reactions represents a major breakthrough in neurodegenerative disease customized treatment.
